# Taking Pain Out of NGF: A “Painless” NGF Mutant, Linked to Hereditary Sensory Autonomic Neuropathy Type V, with Full Neurotrophic Activity

**DOI:** 10.1371/journal.pone.0017321

**Published:** 2011-02-28

**Authors:** Simona Capsoni, Sonia Covaceuszach, Sara Marinelli, Marcello Ceci, Antonietta Bernardo, Luisa Minghetti, Gabriele Ugolini, Flaminia Pavone, Antonino Cattaneo

**Affiliations:** 1 European Brain Research Institute, Rome, Italy; 2 Scuola Normale Superiore, Pisa, Italy; 3 Rottapharm Biotech s.r.l., Trieste, Italy; 4 Institute of Neuroscience, Consiglio Nazionale delle Ricerche, Rome, Italy; 5 Department of Cell Biology and Neurosciences, Istituto Superiore di Sanità, Rome, Italy; The Research Center of Neurobiology-Neurophysiology of Marseille, France

## Abstract

During adulthood, the neurotrophin Nerve Growth Factor (NGF) sensitizes nociceptors, thereby increasing the response to noxious stimuli. The relationship between NGF and pain is supported by genetic evidence: mutations in the NGF TrkA receptor in patients affected by an hereditary rare disease (Hereditary Sensory and Autonomic Neuropathy type IV, HSAN IV) determine a congenital form of severe pain insensitivity, with mental retardation, while a mutation in *NGFB* gene, leading to the aminoacid substitution *R100W* in mature NGF, determines a similar loss of pain perception, without overt cognitive neurological defects (HSAN V). The R100W mutation provokes a reduced processing of proNGF to mature NGF in cultured cells and a higher percentage of neurotrophin secreted is in the proNGF form. Moreover, using Surface Plasmon Resonance we showed that the R100W mutation does not affect NGF binding to TrkA, while it abolishes NGF binding to p75NTR receptors. However, it remains to be clarified whether the major impact of the mutation is on the biological function of proNGF or of mature NGF and to what extent the effects of the R100W mutation on the HSAN V clinical phenotype are developmental, or whether they reflect an impaired effectiveness of NGF to regulate and mediate nociceptive transmission in adult sensory neurons. Here we show that the R100 mutation selectively alters some of the signaling pathways activated downstream of TrkA NGF receptors. NGFR100 mutants maintain identical neurotrophic and neuroprotective properties in a variety of cell assays, while displaying a significantly reduced pain-inducing activity *in vivo* (n = 8–10 mice/group). We also show that proNGF has a significantly reduced nociceptive activity, with respect to NGF. Both sets of results jointly contribute to elucidating the mechanisms underlying the clinical HSAN V manifestations, and to clarifying which receptors and intracellular signaling cascades participate in the pain sensitizing action of NGF.

## Introduction

The neurotrophin Nerve Growth Factor (NGF) [1], [2] was originally identified for its developmental actions, as a neurotrophic survival factor necessary for the development and differentiation of sympathetic and sensory neurons during embryogenesis. In the adult, NGF was subsequently shown to exert pleiotropic actions in various neural and non neural cells, including phenotypic maintenance of basal forebrain cholinergic neurons [3], [4] and functional modulation of sensory neurons [5], [6], [7], [8].

The NGF/TrkA system is known to be a potent mediator of pain [5]. Indeed, NGF is produced in injured tissues and acts a mediator of inflammation [5]. NGF acts directly on peptidergic C fiber nociceptors, which express both NGF receptor tyrosine kinase, TrkA, and the p75 neurotrophin receptor (p75NTR) [6], [9]. TrkA-mediated activation of Erks and PLC−γ1 and p75NTR-mediated c-jun activation have been proposed to contribute to the pronounced pain observed upon NGF administration, *via* the opening of TRPV1 channels [5], [10], [11]. The role of p75NTR in nociception is, on the contrary, more controversial [12], [13]. Also, nothing is known on the relative contribution of NGF versus that of proNGF in the sensitization of nociceptive pathways. Thus, both NGF receptors seem to contribute to peripheral sensitization and nociception, although the extent of their relative contribution remains to be determined.

Besides its actions on the sensitization and hyperexcitability of sensory neurons, NGF exerts a TrkA-mediated chemotactic activity on mast and basophil cells [14], attracting them towards inflammation sites, and inducing their degranulation and secretion of the inflammatory soup [15], [16]. For this reason, the NGF/TrkA system can be considered a master switch for chronic and inflammatory pain responses.

The exogenous administration of NGF induces pain in animals [17], [18] and, when delivered to humans, it induces allodynia, prolonged hyperalgesia, widespread deep pain and muscular tenderness [19], [20], [21]. The pain elicited by NGF infusions was severely dose-limiting, leading to clinical lack of efficacy, in clinical trials for diabetic polyneuropathy [22], [23] or a cause for interruption of Alzheimer's disease trials [24].

The physiological relevance of the NGF system as a crucial regulator of pain [5], [25] is highlighted by robust genetic evidence in humans. Rare forms of congenital insensitivity to pain [human sensory and autonomic neuropathy type and V, HSAN IV (OMIM # 256800) and HSAN V(OMIM # 608654)] are caused by mutations in the *NTRK1* gene, coding for the NGF receptor, TrkA [26], and the NGFB gene [27], [28] respectively. HSAN IV NTRK1 mutations abolish or reduce TrkA responsiveness to NGF [26]. HSAN IV patients show a severe pain insensitivity, anhydrosis and mental retardation, which have been interpreted as due to the developmental consequences of lack of trophic support by the NGF/TrkA system to target neurons, including sensory neurons [26]. Importantly, HSAN V patients, differently from HSAN IV patients, while displaying a similar congenital insensitivity to pain, show no mental retardation nor other neurological and cognitive deficits [29], suggesting that neurodevelopmental effects on NGF target neurons, including sensory neurons, are probably minor in HSAN V patients. The single nucleotide missense mutation in the *NGFB* gene, found in a family of HSAN V patients, who show impaired temperature sensation and an almost complete loss of deep pain perception [27], but normal sweating [30], results in the aminoacid R to W substitution at position 100 of mature NGF protein [27]. The impact of the R100W mutation on NGF functions is unclear [31]. It has been reported that the R100W mutation provokes a reduced processing of proNGF to mature NGF in cultured cells and that the higher percentage of neurotrophin secreted is in the proNGF form [31]. However, it remains to be clarified whether the major impact of the mutation is on the biological function of proNGF or of mature NGF. Also, it is presently unclear to what extent the effects of the R100W mutation on the HSAN V clinical phenotype are developmental, or whether they reflect an impaired effectiveness of NGF to regulate and mediate nociceptive transmission in adult sensory neurons.

For these reasons, we undertook a series of studies to assess the binding and the functional properties of hNGFR100 and hproNGFR100 mutants. In a previous study, we derived hNGF mutants from refolded hproNGF protein, by controlled proteolysis and subsequent chromatography. While all mutants yielded comparable amounts of protein in inclusion bodies, they gave distinct yields after refolding and purification, respectively maximum for wild type hNGF, hNGFR100K and hNGFR100Q, intermediate for hNGFR100A and hNGFR100E and much lower for the genetic mutant hNGFR100W (or for mutants with hydrophobic residues R100Y, R100I, R100L and R100V consistently) [32]. The recombinant proteins were used for *in vitro* receptor binding studies to purified TrkA and p75NTR receptors [32], by surface plasmon resonance (SPR).The hNGFR100 mutants, R100W and R100E, while showing an affinity for TrkA identical to that of hNGF, demonstrated a significantly lower affinity for p75NTR [32]. In the context of hproNGF, the R100W and R100E mutations did not affect at all the binding of hproNGF to TrkA, while the binding of unprocessed hproNGFR100W and hproNGFR100E mutant to p75NTR was not greatly affected, revealing only a two-fold decrease in affinity [32]. Thus, we concluded that, *in vitro*, the major impact of the R100 mutation (notably with the 100W and R100E substitutions) is on the binding of mature hNGF to the p75NTR, while, in the context of unprocessed hproNGF, the mutation has a much lower effect.

The impact of the R100W mutation on receptor binding is consistent with structural predictions based on the crystallographic structures of hNGF complexes with p75NTR [33] and TrkA [34], [35] extracellular domains (Fig. S1A and S1B). Indeed, while hNGF residue R100 is not directly involved in the interface between hNGF and TrkA (Fig. S1A), in the hNGF-p75NTR complex it participates in an extensive charge complementary surface (Fig. S1B). The R100 residue is part of a surface patch involved in an intramolecular interaction of mature hNGF with its pro-domain [32], [36], [37] and therefore the R100W mutation could influence the structure of proNGF and its folding in the closed or extended configurations [36].

In this paper, we assessed the functional properties of hNGFR100 mutants, providing a functional characterization of the R100 mutation in the context of both hNGF and hproNGF. The data show that the R100 mutation, alters the signaling pathways activated downstream of both NGF receptors, abrogating pain-inducing activity, while maintaining identical neurotrophic and neuroprotective properties in cell models. We also show that proNGF has a significantly reduced nociceptive activity, with respect to NGF. These results offer new insights into the mechanisms underlying the clinical manifestations in HSAN V patients, and provide a basis for the development of “painless” hNGF molecules with therapeutic potential for neurodegenerative diseases.

## Results

### Activation of TrkA and p75NTR Signal Transduction Pathways by hNGF Mutants

To characterize the effects of the R100W mutation in cell signaling *in vivo*, a set of mutants, in which the residue R100 was substituted with different amino acids, were expressed in E. coli as hproNGF precursor proteins, and purified to 99% purity, after refolding from inclusion bodies (Table S1), as mature hNGFor unprocessed hproNGF proteins. The recombinant hNGF and hproNGFR100 proteins were used for cell signaling studies in different cell lines.

The activation of TrkA signal transduction pathways by hNGF mutants was studied in BALB/C 3T3-hTrkA cells, in the absence of p75NTR, and in PC12 cells, where p75NTR is also present.

**Figure 1:**
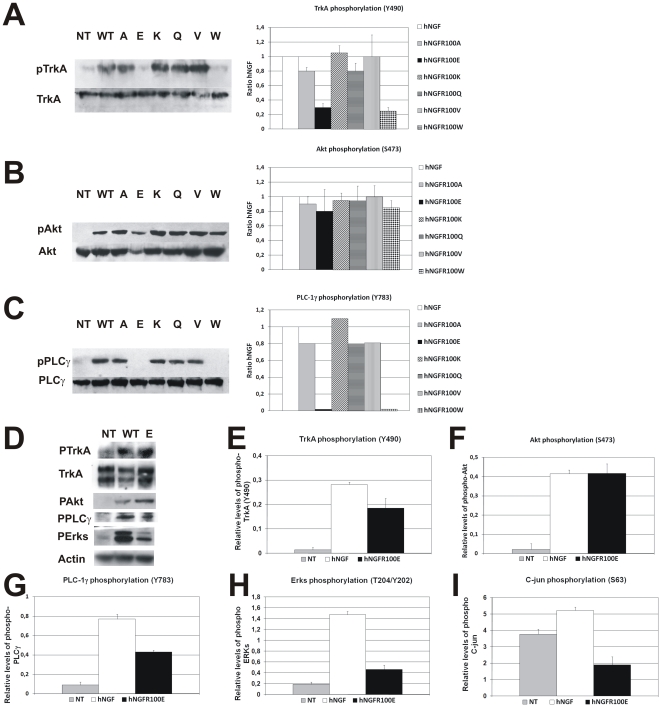
Activation of TrkA and p75NTR signaling by hNGF mutants. Western blot and densitometric analysis of (A) TrkA (Y490), (B) Akt/S473), and (C) PLC-γ1 (Y783) phosphorylation, in extracts from BALB/C 3T3 TrkA cells, stimulated by 100 ng/ml of hNGF and hNGFR100 mutants. D, Western blot of phospho-TrkA (Y490), Akt (S473), PLC-γ1 (Y783) and Erks (T204/Y202) in PC12 cells, stimulated by 5 ng/ml of hNGF and hNGFR100E mutant. E, Densitometric analysis of phospho-TrkA. F, Densitometric analysis of phospho-Akt. G, Densitometric analysis of phospho-PLC-γ1. H, Densitometric analysis of phospho-Erks. I, Densitometric analysis of phospho c-jun in hippocampal neurons. The experiments were performed in triplicate. Bars represent the mean ± s.e.m.

The phosphorylation of residue Tyr490 of TrkA, recruits Shc and activates different downstream cascades, including the Ras/MAP kinase cascade (reviewed in [25], [38], [39], [40], [41] and Fig. S2). The phosphorylation of this residue by hNGFR100W and by hNGFR100E mutants, determined with a site-specific anti- phosphoTrkA antibody, was reduced by 70% with respect to that induced by hNGF, or by other hNGFR100 mutants, in 3T3-TrkA cells (Fig. 1A), but only slightly affected in PC12 cells (Fig. 1D,E). TrkA-dependent signaling linked to neuronal survival is channeled, via the phosphatidylinositol 3-kinase (PI3-K) through the downstream Akt pathway [38], [40]. The activation of Akt in 3T3-TrkA and PC12 cells, by different hNGF proteins, was analyzed with antibodies against active Akt. All hNGFR100 mutants, including R100W and R100E mutants, were equally effective as wild type hNGF in activating this pathway in both cell lines (Fig. 1B,D,F). The phosphorylation of residue Tyr 785 of TrkA, recruits PLC-γ1, inducing its phosphorylation at residue Y783 [38], [40]. In 3T3-TrkA cells, hNGFR100W and hNGFR100E were completely unable to induce the phosphorylation of PLC- γ1 with respect to hNGF (Fig. 1C), unlike other hNGFR100 mutants, confirming that hNGFR100W and hNGFR100E share similar properties. A significant reduction of PLC-γ1 activation by hNGFR100E proteins was also found in PC12 cells (Fig. 1E,G). TrkA activation by NGF leads to the activation of ERK1 and 2 kinases [39], [40]. In PC12 cells the major contribution to overall ERK activation is through RAP-1 and B-Raf, rather than through Ras [42], [43]. The activation of Erks in PC12 cells by hNGFR100E was analyzed with antibodies against active Erks (residues Thr202/Tyr204). The hNGFR100E mutant induced a significantly lower activation of Erks, with respect to hNGF (Fig. 1E,H), which is not due to a different time course (data not shown).

In conclusion, the R100W and R100E mutants, in spite of an overall binding affinity for TrkA that is identical to that of wild type hNGF, show a selective inhibition of certain signaling pathways downstream to TrkA activation. The selective inhibition of TrkA signaling by hNGFR100 mutants occurs both in the absence or in the presence of p75NTR.

Since R100W and R100E hNGF mutants share superimposable receptor binding properties [32] and TrkA signaling properties (Fig. 1), and since the yield of R100E proteins was much higher than that of the R100W mutants, subsequent cell bioassays and *in vivo* studies were performed using the R100E proteins.

As far as p75NTR signaling is concerned, it is expected that the lower binding affinity of hNGFR100E mutants for p75NTR results in a reduction of downstream signaling. The effectiveness of hNGFR100E mutants to activate p75NTR signaling, was assessed by analyzing the phosphorylation of c-jun at residue Ser63, through the p75NTR dependent activation of the jnk kinase [44] (Fig. S2). In hippocampal cells, as expected, the phosphorylation of c-jun by hNGFR100E, was reduced by 30% with respect to that induced by hNGF (Fig. 1I). It is noteworthy that the level of phosphorylation of c-jun induced by hNGFR100E is even lower than the basal levels in untreated hippocampal cells (Fig. 1I).

Thus, mutants hNGFR100W and hNGFR100E differ significantly from hNGF in their ability to activate not only TrkA-dependent signaling pathways, despite their identical binding affinity for TrkA [32] but also p75NTR downstream pathways, with a notable reduction in the ability to activate Erks, PLC−γ1 and c-jun.

### Cellular bioassays with hproNGFR100E and hNGFR100E mutants

Signaling studies confirmed that R100W and R100E hNGF mutants display super imposable properties. Mutants hNGFR100E and hproNGFR100E were therefore chosen also for further studies, aimed at characterizing the impact of the R100 mutation on the NGF and proNGF biological activity in different cellular systems.

**Figure 2:**
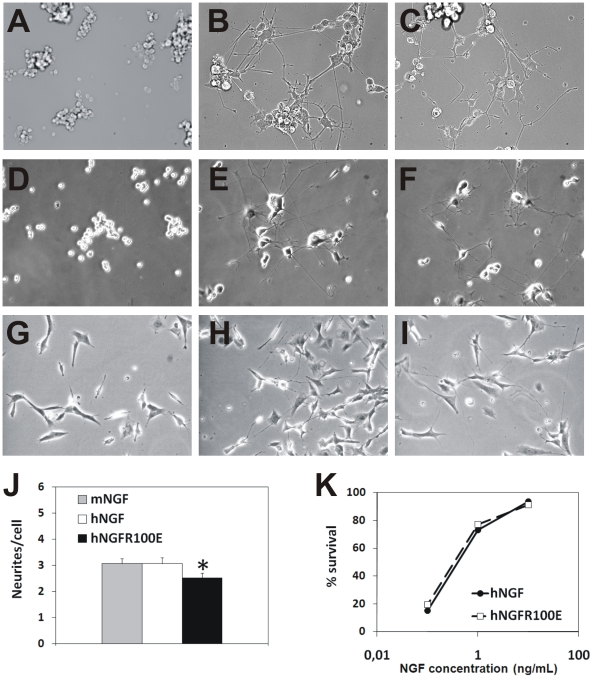
hNGF mutant bioactivity on survival and differentiation of PC12 cells, neuroblastoma SH-SY5Y cells and chick DRG neurons. In Panels A–C, PC12 cells were plated in presence of 100 ng/ml of hNGF (B) or hNGFR100E (C) and the number of PC12 processes evaluated (J). In panels D-F, PC12cells were primed with 50 ng/ml of hNGF (E) or hNGFR100E (F) for 1 week and replated for 2 days in presence of 10 ng/ml of either hNGF or hNGFR100E. Negative controls (A,D) are represented by cells incubated in absence of hNGF or hNGFR100E. (G) Untreated human neuroblastoma SH-SY5Y cells are induced to differentiate when treated for 7 days with 100 ng/ml of hNGF (H), or with hNGFR100E (I). The mutant hNGFR100E is as effective as wild type hNGF in determining the survival and differentiation of chick embryo dorsal root ganglia sensory neurons, after a 48 hrs exposure.

The neurotrophic activity of the R100E mutant was studied, at first, in rat PC12 pheochromocytoma and in human SH-SY5Y neuroblastoma cell lines. The time course and extent of neuronal differentiation of naïve PC12 cells incubated with hNGF or hNGFR100E for one week was by and large identical (Fig. 2A–C, J). Priming of PC12 cells with hNGF or hNGFR100E for one week equally induced NGF dependency upon neurotrophin removal (data not shown), and survival and differentiation of primed rat PC12 cells, induced by 50 ng/ml hNGF and hNGFR100E addition after replating, was identical, both in terms of number of surviving and differentiating cells and of time course and extent of neurite outgrowth (Fig. 2D–F). Also in human SH-SY5Y neuroblastoma cells [45], hNGF, and hNGFR100E were similarly effective in neurite outgrowth induction (Fig. 2G–I).

**Figure 3:**
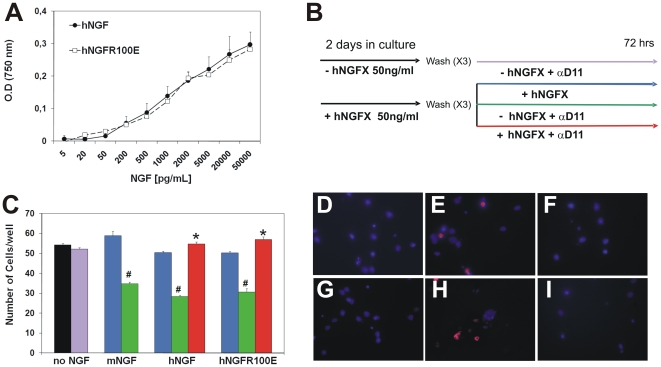
hNGFR100E bioactivity on survival of TF1 and hippocampal neurons. (A) Exposure of human TF1 cells expressing TrkA to hNGF mutants induces a similar proliferative response. (B) Experimental scheme of induction of NGF dependence in rat hippocampal cells and hNGF induced survival. (C) Exposure of rat hippocampal cells to hNGF mutants (HNGFX), for 2 days in culture (priming) induces an NGF dependency (cell death after hNGF removal, green arm), which is rescued by re-exposing the cells to hNGF mutants (blue and red arms). The mutant hNGFR100E is equally effective in inducing dependency after priming and survival of rat hippocampal neurons (total cell counts). (D-I) Representative fields of experimental set-up in B,C. Deprivation of hNGF (E) and of hR100E (H) induces cell death caspase -3 activation (in red), compared to neurons exposed to the respective hNGF mutants (D,G). Exposure to hNGF (F) and hNGFR100E (I) overcomes the cell death induced by anti-NGF addition. Cells were counterstained with 4′,6-diamidino-2-phenylindole (DAPI, in blue).

In order to assess in a more quantitative way the potency of hNGF mutants, the NGF induced proliferation of human erythroleukemia cells TF1 was exploited [46], [47]. In these cells, hNGFR100E induces a dose-dependent proliferation, that is indistinguishable from that induced by hNGF (Fig. 3A). The proliferation index for hNGF and different hNGFR100 mutants are all comprised in a range, between 0.9 and 1.8 ng/ml (Table S2).

The neurotrophic and neuroprotective properties of hNGF and hNGFR100E mutants were then compared in a neuronal amyloidogenic model [48] for neurodegeneration, based on rat hippocampal neuronal primary cultures. In this system hippocampal neurons are, at first, plated for two days in presence of NGF (priming), after which NGF is removed or not (Fig. 3B). Under these experimental conditions, in the presence of 100 ng/ml NGF, hippocampal neurons express increasing (albeit low) levels of TrkA and p75NTR receptors, and constantly high levels of sortilin receptors (Fig. S3). hNGF and hNGFR100E were equally effective in priming hippocampal neurons, thereby inducing NGF dependency, as shown by the extent of neuronal death following removal of the hNGF proteins (hNGFX) and incubation with anti-NGF antibodies (Fig. 3C,E,H). For comparison, naïve rat hippocampal neurons, not “primed” with hNGF, do not acquire this NGF dependency (Fig. 3C). In sister cultures of hNGF and hNGFR100E- primed hippocampal neurons (hNGFX), hNGF and hNGFR100E were shown to be equally effective in overcoming the cell death, induced by NGF deprivation after priming (Fig. 3C, F, I). This experiment demonstrates that the priming, dependency inducing and survival promoting activity of hNGFR100E on hippocampal neurons is identical to that of hNGF.

**Figure 4:**
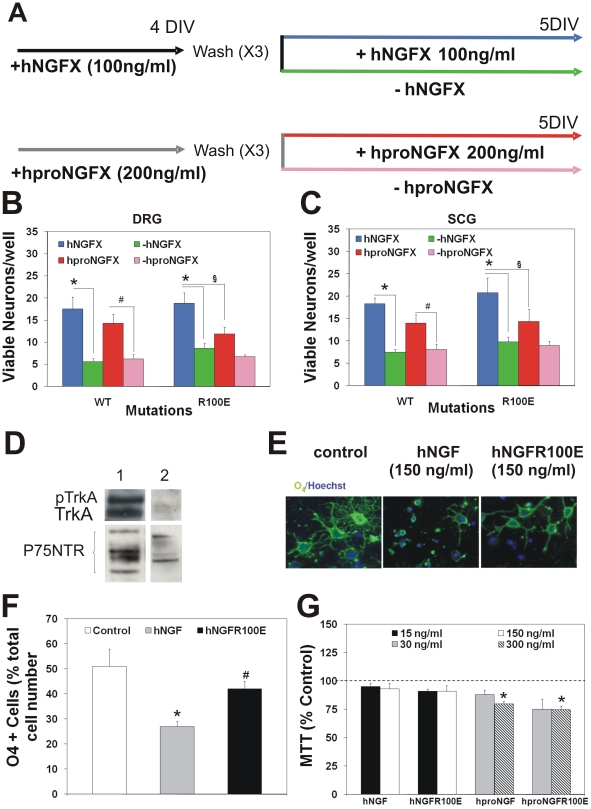
hNGF mutant bioactivity on survival of NGF sympathetic and sensory neurons and oligodendrocyte progenitor cells (OPCs) differentiation. (A), Experimental scheme of induction of NGF dependence in mouse dorsal root ganglia and superior cervical ganglia neurons and hNGF induced survival. The mutant hNGFR100E is as effective as wild type hNGF in determining the survival and differentiation of mouse (B) dorsal root ganglia sensory neurons and (C) superior cervical ganglia, after a 4 days exposure. hproNGFR100E mutant is less effective than hNGF mutants and hproNGF in inducing cells survival in (B) DRGs. (C) hproNGFR100E is less effective in the SCG survival test. (D) Cultured rat OPCs express only the p75NTR receptor. (E) Expression of the oligodendroccyte differentiation marker O4 is reduced in presence of hNGF but not by hNGFR100E. (F) Quantification of the percentage of differentiating OPCs after exposure to hNGF or hNGFR100E. (G) hproNGF and hproNGFR100E induce toxicity in rat OPCs at the dose of 300 ng/ml.

The activity of the hNGF and hproNGF mutants (hNGFX and hproNGFX) was further tested in cultures from mouse dorsal root ganglia (DRG) and superior cervical ganglia (SCG). Cell cultures were first exposed for 4 days to 100 ng/ml or 200 ng/ml of NGF or proNGF, respectively (Fig. 4A). This assay allows to measure both induction of dependency and survival activity of NGF. On the fifth day, DRG and SCG neurons were deprived of hNGFX or hproNGFX for 24 hours, before cell counting (Fig. 4A). In DRG cultures, hNGF and hNGFR100E were similarly effective in determining mouse DRG survival or dependency from NGF (compare blue versus green bars in Fig. 4B). hproNGF was as effective as hNGF in determining neuronal survival (blue versus red bars in Fig. 4B) and dependency (red versus pink bars in Fig. 4B) while hproNGFR100E was less effective than hNGFR100E in inducing DRG survival and dependency (Fig. 4B). Parallel experiments on chick embryo DRG neurons confirmed that survival curves for chick DRG neurons obtained after incubation with different doses of hNGF or hNGFR100E mutant are totally superimposable (Fig. 2K), demonstrating that the neurotrophic potency of hNGFR100E mutants is identical to that of wild type hNGF. In SCG neuronal cultures, we found that, in the context of mature hNGF, the R100E mutation did not affect hNGF ability to induce neuronal survival or dependency (blue versus green bars, Fig. 4C). On the other hand, hproNGFR100E was less effective than hproNGF in inducing SCG survival (blue versus red bars, Fig. 4C) and dependency (red versus pink bars, Fig. 4C).

Therefore, hNGFR100 mutants display a highly effective neurotrophic (survival and dependency) activity in both DRG and SCG cultures. As for hproNGF, in the experimental conditions of the SCG and DG cultures, the neurotrophic effect of proNGF is most likely due to mature NGF molecules cleaved during the incubation with the neurons, and to a reduced cleavage of the hproNGFR100E with respect to hproNGF (data not shown).

The ability of hNGF and hproNGFR100E mutants to activate p75NTR signaling was then evaluated in cultured rat oligodendrocyte progenitors cells (OPCs), which express the p75NTR receptor, in the absence of TrkA (Fig. 4D). hNGF inhibits OPC differentiation ([49] and Fig. 4E,F), while hNGFR100E does not (Fig. 4E,F), confirming that this mutant has a reduced ability to bind p75NTR and activate p75NTR signaling. Conversely, in OPC cultures, hproNGF induces a small but significant amount of cell death, while hNGF does not (Fig. 4G). Unlike the case of hNGFR100E, the mutation R100E, in the context of hproNGF, appears not to affect proNGF activity, since hproNGFR100E mutant induces OPC cell death as effectively as hproNGF (Fig. 4G). On the other hand, hNGFR100E did not affect OPC survival, similarly to hNGF (Fig. 4G).

Thus the OPC culture experiments show that the R100 mutation does indeed impair p75NTR signaling in the context of mature NGF, but does not do so when present in the context of hproNGF. This is in line with the p75NTR binding affinity data (Table S3).

### Effects of hNGF and hproNGF mutants on pain induction

The reduced affinity of hNGFR100 mutants for the p75NTR receptor and their altered TrkA- and p75NTR- mediated signaling properties lead naturally to the question as to whether the hNGFR100 and hproNGFR100 mutants are less effective than hNGF and hproNGF respectively, at triggering a nociceptive response *in vivo*.

**Figure 5:**
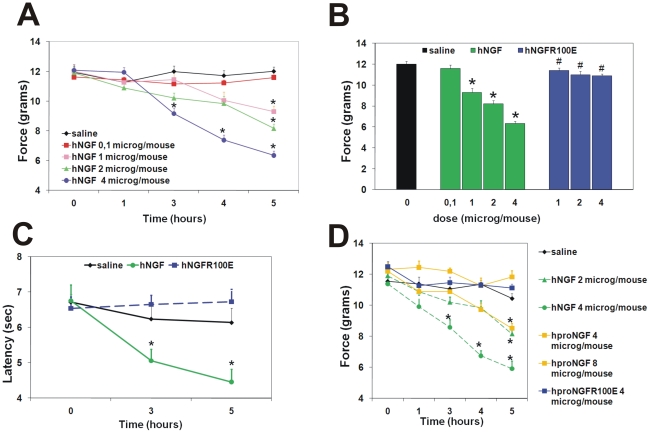
Reduced pro-nociceptive responses of hNGF mutants. (A) Mechanical allodynia: dose-response allodynic effects of hNGF after intraplantar injection in the hindpaw. (B) Mechanical allodynia: reduced allodynic response 5 hours after intraplantar injection of 4 µg/mouse of hNGFR100E compared to hNGF. (C) Thermal hyperalgesic effects in mice injected with hNGFR100E compared to hNGF. (D) Mechanical allodynia: reduced allodynic effects of intraplantar injection of hproNGF versus hNGF, and of hproNGF mutant R100E versus hproNGF. Points represent mean of absolute values ± s.e.m. ANOVA plus post-hoc Tukey-Kramer test; * p<0.001 versus saline, # p<0.01 hNGFR100E versus hNGF.

Mechanical allodynia was measured in adult CD−1 mice exposed to wild type or R100E mutant hNGF by a single injection in the hind-paw. A significant time- and dose-dependent allodynic effect was induced by hNGF (Fig. 5A), as demonstrated by the decreased withdrawal threshold after mechanical stimulation, in the hind-paw ipsilateral to hNGF injection. Controlateral paw showed no significant change in withdrawal thresholds (not shown). Significant allodynia was observed at all doses tested (range  =  0.1–4 µg/injection), except at 0.1 µg/injection, 5 hours after hNGF administration, reaching the maximum for the dose of 4 µg/injection, whose allodynic effect started 3 hours after the injection. As for the hNGFR100E mutant, Fig. 5B shows the paw withdrawal thresholds observed 5 hours after treatments, when the maximal effect by hNGF is observed. hNGFR100E failed to show any allodynic effect, in the dose range tested (Fig. 5B and Fig. S4).

In a thermal hyperalgesia protocol, paw withdrawal latencies were evaluated 3 to 5 hours after hNGF mutant injection into the paw (Fig. 5C). A pronociceptive effect was observed, as expected for hNGF, while, also in this case, no significant reduction of nociceptive threshold whatsoever was observed after hNGFR100E administration, at all time points.

Due to the fact that the R100 mutation could determine a decreased processing of hproNGF to the hNGF [31], thereby determining a relative increase of hproNGF protein, it was of interest to evaluate also the pronociceptive effects of hproNGFR100 mutants. The nociceptive activity of wild type hproNGF was determined at first, since nothing is known on the sensitization of nociceptive pathways by proNGF. A significant allodynic effect was induced by hproNGF at the dose of 4 µg/injection (equimolar to 2 µg/injection of hNGF), but surprisingly, when the dose was doubled (8 µg/injection, equimolar to the hNGF dose that provides the maximum hyperalgesic response, i.e. 4 µg/injection), no nociceptive response at all was observed (Fig. 5D). As for the hproNGFR100E mutant, it failed to show any hyperalgesic effect whatsoever, in the dose range tested (Fig. 5D).

We conclude that both hNGFR100E and hproNGFR100E mutants display a greatly reduced effectiveness in nociceptor sensitization and in eliciting nociceptive responses in mice, with respect to wild type hNGF.

## Discussion

HSANs are a heterogeneous group (I–V) of peripheral neuropathies characterized by sensory and autonomic dysfunctions, involving at least eight different genetic loci (with six identified genes) [50]. A recent study of a large multi-generational Swedish family, suffering from the rare HSAN V form, has led to the identification of a mutation in the NGFB gene (exon 3, nt C661T) [27]. This mutation changes a basic arginine (CGG) to a non-polar tryptophan (TGG) at a position corresponding to residue R100 in mature NGF [27].

HSAN V patients suffer from loss of pain perception but show no mental retardation and have most neurological functions intact [29], suggesting that neurodevelopmental effects on NGF target neurons, including sensory neurons are probably minor in HSAN V patients. The mechanisms whereby the mutant NGFR100W exerts its effects in HSAN V remain however to be investigated.

A recent work [31] showed that, PC12 cells transfected with the cDNA, encoding hproNGFR100W, accumulate unprocessed proNGF and secrete, as a consequence, reduced amounts of mature hNGFR100W. On the basis of that transfection study in one cell line, it was hypothesized that the clinical manifestations of HSAN V may simply depend on a reduced availability of NGF. However, this would not explain the clinical differences between HSAN IV and HSAN V patients, and the reduced neurodevelopmental consequences in the latter, and, moreover, does not address the issue of what is the mechanism for a reduced pain sensation in adult HSAN V patients.

In this work, we exploited a set of well characterized recombinant forms of hNGFR100 mutant proteins [32] to study the functional properties of hNGFR100 mutants, in a variety of cellular systems and *in vivo* pain models, providing a functional characterization of the R100 mutation in the context of both hNGF and hproNGF.

First of all, this study demonstrates that hNGFR100 fails to sensitize and activate nociception, while it shows a full neurotrophic pro-survival competence, providing a direct mechanism for pain insensitivity in HSAN V and explaining the major neurodevelopmental effects in HSAN V. We provide a mechanism for this differential neurotrophic versus nociceptive activity of hNGFR100 mutants. In particular, we show that the interaction between hNGFR100 mutants and TrkA receptor appears to be modified, with respect to that of wild type hNGF, notwithstanding an identical *in vitro* TrkA binding affinity [32]. Indeed, hNGF-R100 mutants differ significantly from hNGF, in their ability to activate downstream TrkA-dependent signaling pathways, with a notable selective reduction in the ability to activate PLC−γ1, while the Akt signaling stream is totally preserved. Understanding how the hNGFR100 proteins bind TrkA with a similar affinity, yet with a different transduction outcome, with respect to hNGF, is a fascinating question that will require detailed structural studies to be understood. Kinetic, rather than equilibrium parameters, might be involved in the transduction mechanism. The demonstrated lower affinity of hNGFR100 for the p75NTR might contribute to the differential outcome of the signaling mediated by TrkA, although the latter was also observed in cells expressing exclusively TrkA, in the absence of p75NTR (Fig. 1). It is remarkable that one single residue mutation confers such a selective alteration in TrkA and p75NTR signaling properties, particularly if compared to previous studies in which a higher number of residues had to be mutated in order to achieve TrkA versus p75NTR selectivity [51] or a TrkA signaling unbalance [52].

In any case, the differential impact of the R100 mutation on the Akt and PLC−γ1, TrkA signaling streams, as well as their reduced p75NTR binding and signaling competence, add new insights into the more general issue of NGF and pain [5], [53].

The specific intracellular signaling mechanism linking TrkA activation to nociception and TRPV1 sensitization still remains to be fully clarified, despite being the subject of intense study, and initial evidence pointing to the activation of PLC−γ1 *via* TrkA [54] was followed by studies implicating the PI3K and MAPK pathways [55]. Our results provide a firm conclusion on the relevance of TrkA- PLC−γ1 signaling in nociception sensitization.

The role of p75NTR in pain is still controversial [12], [13]. Although the general consensus is that most NGF actions on pain transmission and sensitization, are mediated by TrkA, growing evidences suggest that p75NTR also contributes. A number of p75NTR-mediated signaling pathways activated by NGF have been suggested to mediate peripheral sensitization, independently of TrkA activity (reviewed by Nicol and Vasko [25] and schematically illustrated in Fig. S2). However, NGF-induced pain has been shown to occur in p75NTR −/− knock-out mice, although these mice are less sensitive to heat and mechanical stimulation [13]. On the other hand, p75NTR has been associated with NGF-induced excitability of nociceptors in culture [56], with pain states in which bradykinin is an important mediator [57]. Moreover p75NTR functional block has been shown to suppresses injury-induced neuropathic pain [12] and the hyperalgesia arising from complete Freund's Adjuvant-induced inflammation or from an intraplantar injection of NGF [58]. In this scenario, on one hand our work demonstrates a significant contribution of p75NTR signaling to the nociceptive actions of NGF, since the R100 mutants displays a greatly reduced binding to p75NTR. However, the relative contribution of the TrkA signaling unbalance and of the p75NTR reduced binding in determining the failure of hNGFR100 to induce pain is likely to be complex. Indeed, we found that, unexpectedly, the preferred p75NTR receptor ligand proNGF has a reduced capacity to sensitize and activate nociception, compared to mature hNGF. Introducing the R100 mutation further reduced the ability of hproNGF to induce pain. This suggests that p75NTR receptor activation *per se* is not the crucial sensitizing event, and, conversely, that it is not abolishing p75NTR signaling per se that is responsible for the failure of hNGFR100 to induce pain.

In a number of different survival, differentiation and proliferation cellular assays, NGFR100 mutants showed no difference with respect the hNGF counterparts. The observation that neurotrophic properties are not affected by the R100 mutation may explain why in HSAN V patients developmental deficits affecting CNS appear very limited or absent. On the other hand, the ability of NGFR100 mutants to induce pain sensitization in animal models was markedly reduced, correlating well with the loss of pain perception in HSAN V patients. On the whole, these results provide an explanation for the clinical impact of the NGFR100 mutation, showing that the survival functions of NGF in neuronal development are largely unaffected by this mutation. Differentiation of nociceptors by NGF R100 mutants was not determined in the present study, so an effect on nociceptor differentiation could contribute to the limited neurodevelopmental loss of sensory Aδ and C fibers observed in some HSAN V patients [59]. However, the results show that the main impact of the R100 mutation is on a great reduction of the pain-sensitizing functions of NGF, after neuronal development has been completed. Thus, the molecular explanation for the HSAN V hNGFR100W mutation lies in an alteration of the separate signaling streams normally activated by NGF through its receptors. hNGFR100 proteins maintain the neurotrophic signaling stream unchanged, while showing an impairment of the p75NTR- and TrkA-mediated signaling involved in nociceptor sensitization. In light of the unchanged prosurvival properties of hNGFR100, the clinical phenotype in HSAN V patients could be therefore determined by a lower hNGFR100 versus hproNGFR100, resulting from impaired secretion or processing [31] (possibly involving an altered intramolecular interaction of the R100 surface patch with the pro-domain[32]), as well as by a reduced pain sensitizing activity of hNGFR100 and hproNGFR100. Therefore, at least two concomitant mechanisms might determine the clinical phenotype of HSAN V patients: a reduced processing/secretion efficiency [31], and a signaling unbalance selectively affecting the nociceptive regulatory actions (and possibly nociceptor differentiation), while preserving the pro-survival ones, by hNGF R100. Further experiments are required to demonstrate whether and how the R100 mutation affects some aspects of the differentiation of sensory nociceptive neurons.

In this respect, another recently described V232fs mutation in the *NGFB* gene, purportedly linked to HSAN V [28], leads to a clinical picture characterized by inability to perceive pain, mental retardation and anhydrosis. This would appear, instead, clinically more similar to a HSAN IV phenotype. Consistently, this V232fs frameshift mutation determines a NGF protein in which the terminal 15 aminoacids are replaced with a novel 43 aminoacid terminal sequence, resulting in a functionally null protein [28].

The fact that the NGF mutation R100W appears, from a clinical point of view, to separate the effects of NGF on CNS development from those involved in the activation of adult peripheral pain pathways, could provide a basis for designing “painless” NGF variant molecules, tailored for therapeutic applications in Alzheimer's disease [60], circumventing the most serious hurdle that have limited such applications. More generally, these results could set the basis for a designer neurotrophin for those applications where targeting selectively TrkA pathways, without the confounding actions associated to p75NTR, would be advantageous.

## Methods

### hNGF and hproNGF mutants expression and purification

Mutagenesis, hNGF mutants expression and purification were performed as previously described [32].

### *In vitro* phosphorylation assays

BALB/C 3T3- hTrkA cell transfectants (3T3 hTrkA cells, expressing 10^6^ human TrkA per cell, kindly provided by Stefano Alema CNR Institute of Cell Biology, Roma, Italy) PC12 cells [61] and hippocampal neurons [62] were used to assess NGF receptor activation, as well as downstream signaling, after incubation in the presence of 100 ng/ml (for 3T3-TrkA and hippocampal neurons) or 5 ng/ml (for PC12 cells) of hNGF or hNGF mutants. Details of the assays are reported in **Methods S1**

### *In vitro* survival, proliferation and neurotrophic assays

PC12 cells were plated in presence of 100 ng/ml of hNGF or hNGF mutants. Alternatively, PC12 cells were primed with hNGF or hNGF mutants (50 or 100 ng/ml of NGF for 1 week) and then replated in the presence or absence of 10–50 ng/ml hNGF or hNGF mutants as described [63]. TF1 cells (ATCC-LGC Standards, Teddington, UK) assay was performed as described [47]. Chick embryonic DRGs (E6–E9) were collected, cleaned, trypsinized, dissociated and cultured in the absence or presence of hNGF mutants (0,1–10 ng/ml) as described [64]. Human neuroblastoma SH-SY5Y cells (ATCC) and rat hippocampal neuron assays were performed according to LoPresti et al [65] and Matrone et al. [48], respectively. In the rat hippocampal neuron model, neuronal death following NGF removal is directly caused by aberrant APP processing [48], and thus represents a cellular model directly linking neurotrophic deficits and Alzheimer's neurodegeneration. Rat oligodendrocytes were isolated and incubated with the different neurotrophins as described before [66]. Details are provided in **Methods S2**.

### *In vivo* nociceptive assays

CD1 male mice, weighing 40–45 g, from Charles River Labs (Como, Italy) were used for nociceptive tests. Different groups of mice were used for mechanical allodynia and thermal hyperalgesia behavioral testing. The pro-nociceptive inducing activity of hNGF was compared to that of hNGFR100E mutant, hproNGF and hproNGFR100E mutant. To this purpose, since the expression yield for the genetic mutant R100W was too low to carry out *in vivo* experiments [32], the nociceptive tests were performed with the functionally equivalent R100E mutants. Mice were intraplantarly (i.pl.) injected, on their hindpaws' plantar surface, with 20 µl of hNGF or of hNGFR100E at concentrations corresponding to 1, 2 and 4 µg/20 µl/mouse in saline 0.9% NaCl. For hproNGF and hproNGFR100E, experiments were performed at equimolar concentrations to the hNGF, corresponding to 4 and 8 µg/20 µl/mouse. Control mice were injected with 20 µl of saline. Behavioral measurements were made 1 hour before (baseline) and 1, 3, 4 and 5 hours after i.pl. injections, for mechanical allodynia and 5 hours after i.pl. injections for thermal hyperalgesia.

Mechanical allodynia was quantified as paw withdrawal threshold in response to a mechanical stimulus of increasing strength, using the Dynamic Plantar Aesthesiometer (,Ugo Basile, Italy). The apparatus is an automated von Frey system with the cutoff force set at 20 grams, as previously described [67], [68]. Animals were placed in plastic cages with a wire net floor, 5 min before the experiment. The mechanical stimulus (a slight pressure to the skin) was applied to the midplantar surface of the hind paw, as described [68]. At each testing day, the withdrawal thresholds in the paws ipsi- and contra-lateral to the injection were taken as the mean of three consecutive measurements per paw, with 10-s interval between each measurement.

Thermal hyperalgesia was assessed in mice using the Plantar test (Plantar Test, Basile, Italy). The pain threshold was determined measuring the paw withdrawal latency to a thermal stimulus constituted by a beam of I.R. source, focused through the glass floor onto the plantar surface of the paw, until the animal lifted the paw away.

All experiments were conducted according to national and international laws for laboratory animal welfare and experimentation (EEC Council directive 86/609, OJ L 358, 12 December 1987. Experimentation was approved by Italian Department of health (approval n. 9/2006).

### Statistical Analyses

Statistical analyses were performed using the Sigmastat v. 3.11 program (Systat Software, San Jose, CA). The alpha was set at 0.05 and a normality and equal variance test were first performed.

All values of behavioral tests to assess nociception are expressed as mean ± s.e.m of 8–10 animals per group. Two-way ANOVAs for repeated measures were used to analyse the effects of pharmacological treatments. Post-hoc comparisons were carried out using Tukey-Kramer test. Differences were considered significant at p<0.05.

## Supporting Information

Methods S1 Details of in vitro phosphorylation assays.
(DOC)

Methods S2 Details of *in vitro* survival, proliferation and neurotrophic assays
(DOC)

Table S1 List of NGF from different species and muteins derived from hNGF
(DOC)

Table S2 Concentration of hNGFR100 mutants necessary to achieve half-maximum (50%) TF1 cell proliferation (dose range 5–50,000 pg/ml)
(DOC)

Table S3 Summary of the derived kinetic and equilibrium binding constants of hproNGF and hNGF and their muteins in position 100 towards TrkA and p75 receptors
(DOC)

Figure S1 **Structural insights into the R100W HSAN V mutation in NGFB protein.** The crystallographic structures of hNGF (in blue) complexed with TrkA (A) and with p75NTR (B) extracellular domains show that hNGF residue R100 (in green) is not directly involved in the interface between hNGF and TrkA (A), while (B) it participates in the hNGF-p75NTR interaction surface. Cartoon representations created with *Pymol* (http://www.pymol.org).
(TIF)

Figure S2 **hNGF activation of TrkA and p75 NTR and their associated intracellular signaling pathways.** The cartoon illustrates in a schematic manner the activation of TrkA and p75NTR by hNGF and the main downstream signaling pathways. As shown, the signaling streams leading to pain or to survival and growth/differentiation involve largely distinct signaling molecules, downstream of TrkA and p75NTR. Modified from Nicol and Vasko [25].
(TIF)

Figure S3 **Expression of NGF receptors TrkA, P75NTR and sortilin in hippocampal neurons.** Western blot and densitometric analysis of (A) pTrkA (Y490) (B) p75NTR and (C) sortilin in cell extracts from hippocampal cells after 3 and 5 days of culture compared to PC12 cells. Hippocampal cells were stimulated with 4 nM NGF.
(TIF)

Figure S4 **Time course of dose-dependent nociceptive response triggered by hNGF muteins.** (A) 1 µg/mouse; (B) 2 µg/mouse and (C) 4 µg/mouse. At all doses and time points hNGFR100E does not induce pain. Points are the mean of the percentage derived from the ratio between ipsilateral vs controlateral measures ± s.e.m.
(TIF)
