# Supplementary material for: Taking Pain Out of NGF: A “Painless” NGF Mutant, Linked to Hereditary Sensory Autonomic Neuropathy Type V, with Full Neurotrophic Activity
Source: PLoS One. 2011 Feb 28;6(2):e17321. doi: 10.1371/journal.pone.0017321 (PMC3046150; doi:10.1371/journal.pone.0017321)
Supplement: Methods S1 — Details of in vitro phosphorylation assays. (DOC) [file pone.0017321.s001.doc]

**Supplementary Methods S1**

**In vitro TrkA and p75NTR downstream signaling.** BALB/C 3T3-transfected cells (3T3 TrkA cells), expressing 10^6^ human TrkA molecules per cell (kindly provided by Stefano Alema Institute of Cell Biology, Consiglio Nazionale delle Ricerche (CNR), Roma, Italy), were maintained in DMEM medium (Invitrogen Corp., Carlsbad, CA, USA), supplemented with 10% fetal calf serum. Briefly, 3T3 TrkA cells were plated in 35 mm Petri dishes at a density of 10^6^ cells per dish. The day after, 3T3 TrkA cells were starved with serum-free medium supplemented with 0.05% BSA for 1 h at 37°C. Thereafter, serial equimolar dilutions of wild type hNGF and R100 mutants were incubated for 10 minutes at 37°C with the cells. After PBS wash, cells were scraped in cold RIPA buffer supplemented with phosphatases and proteases inhibitors (50mM Tris pH 7.4, 150mM NaCl, 1% Triton X100, 1% Na deoxycholate, 10mM EDTA, protease inhibitor cocktail purchased by Roche, 1 mM sodium orthovanadate, 50 mM NaF, 100 nM okadaic acid); insoluble material was removed with a 5 min, 10 000 rpm centrifugation. The extracts were quantified using the Lowry test (Bio-Rad) and standardized in the subsequent assays. Rat PC12 pheochromocytoma cells[1] were maintained in RPMI 1640 medium (Invitrogen), supplemented with 5% fetal calf serum and 10% heat-inactivated horse serum. To evaluate the activation of the different signaling pathways cells were incubated for 30 minutes in the presence of 5 ng/ml of the corresponding mutant. Hippocampal neurons were prepared from embryonic day 17–18 (E17-18) embryos from timed pregnant Wistar rats (Charles River Laboratories Italia, Calco, Italy). The hippocampus was dissected out in Hanks’ balanced salt solution buffered with Hepes and dissociated *via* trypsin/EDTA treatment. Cells were plated at 1 x 10^6^ cells on 3.5-cm dishes precoated with poly-DL-lysine (conc.). After 2 days of culturing in Neurobasal medium with B-27 supplement and glutamax (Invitrogen), cytosine arabinofuranoside was added to reduce glial proliferation. Half of the medium was changed every 3–4 days. Neurons were incubated by exposing them to hNGF or hNGF mutants (100 ng/ml) for 30 min 3-5 days after plating. PC12 cells and hippocampal neurons were lysed in RIPA buffer as described above.

*Western blot assay* Extracts of 3T3 TrkA (from cells treated with 50, 100 and 260 ng/ml of hNGF or hNGF mutants) were obtained as described above, separated on SDS polyacrylamide 10% gels and transferred to nitrocellulose. After blocking in MPBS (5% nonfat dry milk(PBS) for 1 h at room temperature with gentle agitation , the filters were incubated over night at 4°C with the anti-phosphoserine 476 Akt antibody (Cell Signaling Technology, diluted 1:1000), the anti-Akt antibody (Cell Signaling Technology, diluted 1:1000), the anti-phosphotyrosine 490 TrkA antibody (Cell Signaling Technology, diluted 1:1000), the anti−phosphotyrosine 783 PLC1γ antibody (Cell Signaling Technology, diluted 1:1000)and the anti-TrkA antibody (R&D Systems Inc., diluted 1:1000), followed respectively by anti-rabbit antibody for the first four and, for the latter, by the anti-mouse secondary antibody, coupled to horseradish peroxidase HRP (diluted 1:1000) for 1h at room temperature. After washes in PBST (0.1% Tween 20) and PBS at room temperature with gentle agitation, the HRP conjugates were detected with the electrochemiluminescence protocol (GE Healthcare Life Sciences, Amersham, UK).

1. Greene LA, Tischler AS (1976) Establishment of a noradrenergic clonal line of rat adrenal pheochromocytoma cells which respond to nerve growth factor. Proc Natl Acad Sci U S A 73: 2424-2428.
