# Supplementary material for: Taking Pain Out of NGF: A “Painless” NGF Mutant, Linked to Hereditary Sensory Autonomic Neuropathy Type V, with Full Neurotrophic Activity
Source: PLoS One. 2011 Feb 28;6(2):e17321. doi: 10.1371/journal.pone.0017321 (PMC3046150; doi:10.1371/journal.pone.0017321)
Supplement: Methods S2 — Details of in vitro survival, proliferation and neurotrophic assays (DOC) [file pone.0017321.s002.doc]

**Supplementary Methods S2**

**TF1 cell proliferation assay.** TF1 cells were cultured for 1 week in RPMI 1640 medium with 2 mM L-glutamine adjusted to contain 1.5 g/l sodium bicarbonate, 4.5 g/l glucose, 10 mM HEPES, and 1 mM sodium pyruvate (90 %), supplemented with 2 ng/ml recombinant human GM-CSF (R&D Systems Inc.), and 10% fetal bovine serum.

The TF1 proliferation assay was performed in 96 wells microtiter plates by incubating 15,000 cells per well in the presence of several doses of either wild type hNGF and corresponding R100 mutants ranging between 50,000 and 5 pg/ml. Cells were seeded 1h before adding treatments. A MTT Cell proliferation assay (ATCC kit) was employed to evaluate cell response: after a 40-hour culture period, MTT solution was added for an additional 4 hours incubation, according to the previously described colorimetric assay [1]. The intensity of each colorimetric signal was measured at 570 nm in a microtiter plate reader, 16 hours (overnight) following addition of detergent reagent. In all experiments, each different treatment was done in triplicate.

**SH-S5SY and PC12 cell differentiation assays .** PC12 cells were maintained in RPMI 1640 medium (Invitrogen), supplemented with 5% fetal calf serum and 10% heat-inactivated horse serum. cells were primed with 100 ng/ml of the different hNGF mutants for 1 week and replated in the presence of 50 ng/ml of the corresponding mutant. The assay was terminated 4 days later.

SH-S5SY Cells were grown in DMEM/F12 Glutamax medium supplemented with 10% heat-inactivated fetal bovine serum, 2 mM glutamine, and 100 pg/ml gentamicin. For differentiation studies, cells were plated (1.5 x 10^4^ to 5 x 10^5^ cells/dish) in 35-mm Falcon dishes. After 5 days of 0.3 μM aphidicolin treatment, cells were allowed to differentiate for 7 days in presence of 100 ng/ml of hNGF or hNGF mutants.

**Hippocampal cell assay.** In this system, rat hippocampal neurones are rendered NGF-dependent by incubation with NGF for two days in culture, after which, upon removal of NGF and anti-NGF addition, cells activate aberrant amyloidogenesis that causes Aβ peptide-dependent cell death [2]. In this system, neuronal death following NGF removal is directly caused by aberrant APP processing[2] Hippocampal neurons were prepared from embryonic day 17–18 (E17-18) embryos from timed pregnant Wistar rats (Charles River Laboratories Italia, Calco, Italy) . The hippocampus was dissected out in Hanks’ balanced salt solution buffered with Hepes and dissociated *via* trypsin/EDTA treatment. Cells were plated at 1 x 10^6^ cells on 3.5-cm dishes precoated with poly-DL-lysine (conc.). After 2 days of culturing in Neurobasal medium with B-27 supplement and glutamax (Invitrogen), cytosine arabinofuranoside was added to reduce glial proliferation. Half of the medium was changed every 3–4 days. Neurons were primed by exposing them to hNGF or hNGFR100E (50 ng/ml) for 48 h at 3–4 days after plating, washed 3 times with the medium and incubated for additional 48 h in presence of the anti-NGF monoclonal antibody αD11[3] (10 μg/ml) or in the presence of αD11 and the same mutant (50 ng/ml) that was used during the priming. Viable hippocampal neurons were quantified by counting the number of intact nuclei. The presence of cell death was revealed by active caspase-3 immunoreactivity ,incubating cells with the anti-active caspase 3 antibody (1:100, Cell Signalling technology). Cells were counterstained with 4',6-diamidino-2-phenylindole (DAPI, in blue).

**DRG and SCG neurons survival assay**.

Superior cervical ganglia and dorsal root ganglia were dissected from postnatal day 3 C57BlxSJL mice, enzymatically dissociated, and plated on 48 well cells culture dishes. Neurons were maintained in growth medium consisting of DMEM supplemented with fetal bovine serum (10%), penicillin (1 U/ml), streptomycin (1 U/ml), and hNGF (100 ng/ml), hNGFR100E (100 ng/ml), hproNGF (200ng/ml) or hproNGFR100E (200 ng/ml). After 4 days, a subset of neurons were washed and incubated in culture medium without the addition of neurotrophins for 48 hours. Neurons were lysed and cell counts were performed.

**Oligodendrocytes cultures and differentiation assay.** Purified cultures of OPC were obtained as previously described [4], with minor modifications. Briefly, primary cultures were prepared from forebrain of postnatal day 1 Wistar rats (Harlane, Milan, I) by mechanical dissociation. Cells were grown on poly-L-lysine coated 60-mm-diameter culture dishes in Dulbecco’s modified Eagle’s medium (DMEM with high glucose) supplemented with 10% fetal bovine serum. After 10 days, a cell population enriched in OPC was mechanically detached from the mixed cultures and incubated for 1 h at 37°C in culture flasks to remove adhering cells and thus minimize contamination by microglial cells. Cells were then seeded at the density of 6 x 10^4^ cells/cm^2^ into poly-L-lysine-coated 35 or 60-mm diameter plastic culture dishes or 96 well plates. This method yields an almost pure population (>98%) of cells of the oligodendrocyte lineage, as assessed by immunofluorescence staining with LB_1_, O_4_, and O_1_ antibodies, which bind to differentiation-regulated antigens of these cells. Astrocytes, microglia, and fibroblasts accounted for less than 2% of total cells, as assessed by cell type specific markers, whereas neurons were absent. At 2 h after plating, the culture medium was replaced with a chemically defined serum-free medium, consisting of DMEM/Ham F12 (4:1) supplemented with 5.6 mg/ml glucose, 5 µg/ml insulin, 100 µg/ml human transferrin, 100 µg/ml bovine serum albumin, 0.06 ng/ml progesterone, 40 ng/ml sodium selenite, 16 µg/ml putrescine, 50 U/ml penicillin, 50 µg/ml streptomycin and 2mM glutamine. Cell culture reagents were from Invitrogen (Milan, I); other chemicals were form Sigma-Aldrich Italia (Milan, I). After 18h, cell were added with hNGF (15ng/ml), hNGF100E (150 ng/ml), hproNGF (30ng/ml) or hproNGF100E (300 ng/ml) and incubated for further 24h.

# Immunocytochemistry and viability test. Cells were characterize for developmental-dependent antigen expression by using the monoclonal antibody O_4_ (mouse IgM, hybridoma supernatant, diluted 1:3), as previously described [4]. As secondary antibodies, fluorescein-conjugated goat anti-mouse IgM were used. Cells were then fixed in 4% paraformaldehyde for 15 min at RT. Total cell number was assessed by staining the cultures with Hoechst 33258 vital dye, added during the last hour of incubation. Coverslips were mounted with Vectashield Mounting Medium (Vector Laboratories, Burlingame, CA). Cells were counted in 10 microscopic fields of 0.18 mm^2^ per coverslip using a Leica DM4000B fluorescence microscope equipped with DFC420C digital camera and Leica Application Suite Software (260RI) for image acquisition. An average of 100 cells per field was counted at least in two coverslips for each condition in at least three independent experiments examined. An observer blind to treatment conditions conducted all analyses.

Cell viability was assessed by 3-(4,5-dimethyl thiazol-2-y1)-2,5-diphenyl tetrazolium bromide (MTT, Sigma-Aldrich Italia, Milan, I), as previously described [4]. MTT was added for the last 4 h of incubation. The medium was then removed and 100 µl of DMSO added to each well to dissolve the dark blue crystals. Microplates were then read on GDV programmable microplate reader, using a test wavelength of 570 nm and a reference wavelength of 630 nm.

1. Mosmann T (1983) Rapid colorimetric assay for cellular growth and survival: application to proliferation and cytotoxicity assays. J Immunol Methods 65: 55-63.

2. Matrone C, Ciotti MT, Mercanti D, Marolda R, Calissano P (2008) NGF and BDNF signaling control amyloidogenic route and Abeta production in hippocampal neurons. Proc Natl Acad Sci U S A 105: 13139-13144.

3. Cattaneo A, Rapposelli B, Calissano P (1988) Three distinct types of monoclonal antibodies after long-term immunization of rats with mouse nerve growth factor. J Neurochem 50: 1003-1010.

4. Bernardo A, Greco A, Levi G, Minghetti L (2003) Differential lipid peroxidation, Mn superoxide, and bcl-2 expression contribute to the maturation-dependent vulnerability of oligodendrocytes to oxidative stress. J Neuropathol Exp Neurol 62: 509-519.
