# Supplementary material for: Taking Pain Out of NGF: A “Painless” NGF Mutant, Linked to Hereditary Sensory Autonomic Neuropathy Type V, with Full Neurotrophic Activity
Source: PLoS One. 2011 Feb 28;6(2):e17321. doi: 10.1371/journal.pone.0017321 (PMC3046150; doi:10.1371/journal.pone.0017321)
Supplement: Table S1 — List of NGF from different species and muteins derived from hNGF (DOC) [file pone.0017321.s003.doc]

Supplementary Table 1. List of NGF from different species and muteins derived from hNGF.

|  | **denomination** | **Sequence** |
| --- | --- | --- |
| Mouse NGF | mNGF | **ID.SEQ.N.1**  **SWISS PROT accession number: P01139** |
| Human NGF | hNGF | **ID.SEQ.N.3**  **SWISS PROT accession number: P01138** |
| Mouse -proNGF | m-proNGF | **ID.SEQ.N.4**  **SWISS PROT accession number: P01139** |
| Human -proNGF | h-proNGF | **ID.SEQ.N.5**  **SWISS PROT accession number: P01138** |
| Human NGF mutated in position 100 | hNGFR100W | **ID.SEQ.N.7**  AAW**W**FIR |
| Human NGF mutated in position 100 | hNGFR100A | **ID.SEQ.N.8**  AAW**A**FIR |
| Human NGF mutated in position 100 | hNGFR100E | **ID.SEQ.N.9**  AAW**E**FIR |
| Human NGF mutated in position 100 | hNGFR100K | **ID.SEQ.N.10**  AAW**K**FIR |
| Human NGF mutated in position 100 | hNGFR100Q | **ID.SEQ.N.11**  AAW**Q**FIR |
| Human NGF mutated in position 100 | hNGFR100V | **ID.SEQ.N.12**  AAW**V**FIR |
| Human-pro-NGF mutated in position 100 | h-proNGFR100E | **ID.SEQ.N.18**  AAW**E**FIR |
