# Supplementary material for: Taking Pain Out of NGF: A “Painless” NGF Mutant, Linked to Hereditary Sensory Autonomic Neuropathy Type V, with Full Neurotrophic Activity
Source: PLoS One. 2011 Feb 28;6(2):e17321. doi: 10.1371/journal.pone.0017321 (PMC3046150; doi:10.1371/journal.pone.0017321)
Supplement: Table S2 — Concentration of hNGFR100 mutants necessary to achieve half-maximum (50%) TF1 cell proliferation (dose range 5–50,000 pg/ml) (DOC) [file pone.0017321.s004.doc]

Supplementary Table 2. Concentration of hNGFR100 mutants necessary to achieve half-maximum (50%) TF1 cell proliferation (dose range 5-50,000 pg/ml)

| **Mutants** | **Concentration corresponding to 50% cell proliferation (ng/ml)** |
| --- | --- |
| **hNGF** | 1.80 |
| **hNGFR100A** | 4.27 |
| **hNGFR100E** | 1.28 |
| **hNGFR100K** | 0.92 |
| **hNGFR100Q** | 1.11 |
