# Supplementary material for: Taking Pain Out of NGF: A “Painless” NGF Mutant, Linked to Hereditary Sensory Autonomic Neuropathy Type V, with Full Neurotrophic Activity
Source: PLoS One. 2011 Feb 28;6(2):e17321. doi: 10.1371/journal.pone.0017321 (PMC3046150; doi:10.1371/journal.pone.0017321)
Supplement: Table S3 — Summary of the derived kinetic and equilibrium binding constants of hproNGF and hNGF and their muteins in position 100 towards TrkA and p75 receptors (DOC) [file pone.0017321.s005.doc]

**Table 3** **SPR analysis.** Summary of the derived kinetic and equilibrium binding constants of hproNGF and hNGF and their muteins in position 100 towards TrkA and p75 receptors.

| **TrkA** | | | | |
| --- | --- | --- | --- | --- |
|  | Ka (M^-1^s^-1^) | Kd (s^-1^) | KD (M) | KA (M^-1^) |
| hproNGF | 6,63 10^4^ | 1,18 10^-3^ | 17,8 | 5,62 10^7^ |
| hproNGF R100E | 4,21 10^4^ | 0,86 10^-3^ | 20,6 | 4,86 10^7^ |
| hNGF | 1.23 10^6^ | 1.16 10^-3^ | 0.94 | 1.06 10^9^ |
| hNGFR100E | 1.34 10^6^ | 1.93 10^-3^ | 1.44 | 6.9 10^8^ |
| **p75NTR** | | | | |
|  | ka | kd | KD | KA |
| hproNGF | 2,3910^5^ | 4,72 10^-3^ | 19,7 | 5,07 10^7^ |
| hproNGF R100E | 4,18 10^4^ | 2,2 10^-3^ | 52,5 | 1,9 10^7^ |
| hNGF | 3.05 10^6^ | 4.68 10^-3^ | 1.53 | 6.52 10^8^ |
| hNGFR100E | 4.51 10^4^ | 5.62 10^-3^ | 125  | 8.02 10^6^ |
