# Supplementary figures and images for: Taking Pain Out of NGF: A “Painless” NGF Mutant, Linked to Hereditary Sensory Autonomic Neuropathy Type V, with Full Neurotrophic Activity
Source: PLoS One. 2011 Feb 28;6(2):e17321. doi: 10.1371/journal.pone.0017321 (PMC3046150; doi:10.1371/journal.pone.0017321)

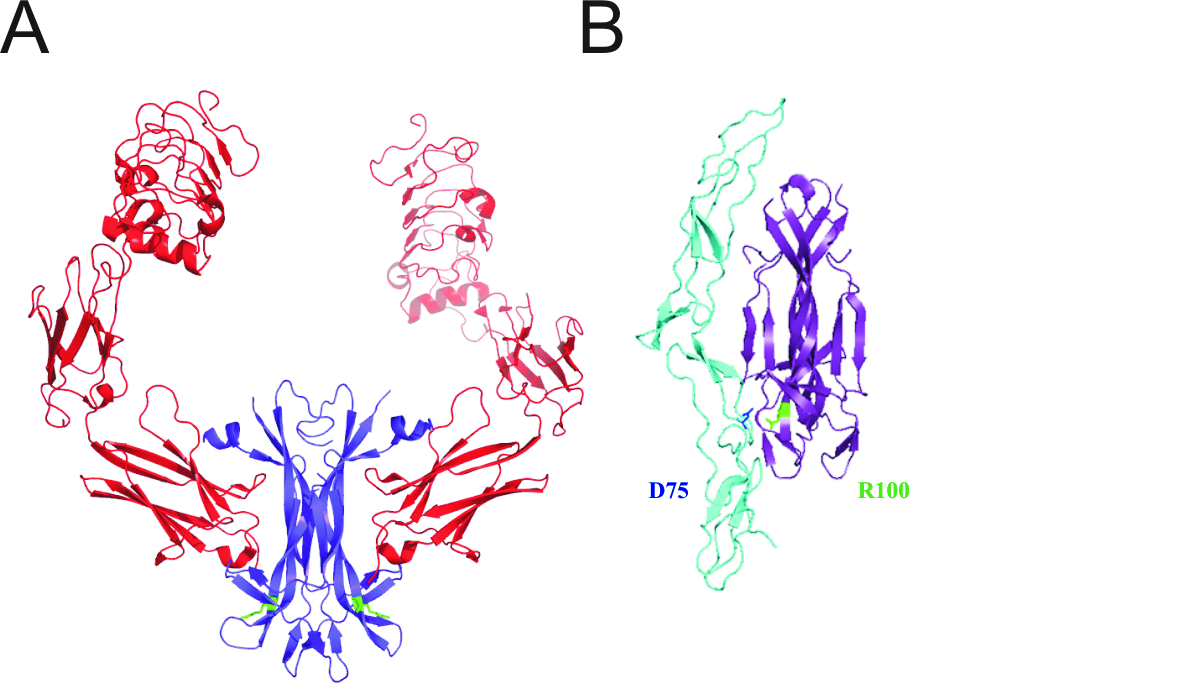

Supplement: Figure S1 — Structural insights into the R100W HSAN V mutation in NGFB protein. The crystallographic structures of hNGF (in blue) complexed with TrkA (A) and with p75NTR (B) extracellular domains show that hNGF residue R100 (in green) is not directly involved in the interface between hNGF and TrkA (A), while (B) it participates in the hNGF-p75NTR interaction surface. Cartoon representations created with Pymol (http://www.pymol.org). (TIF) [file pone.0017321.s006.tif]

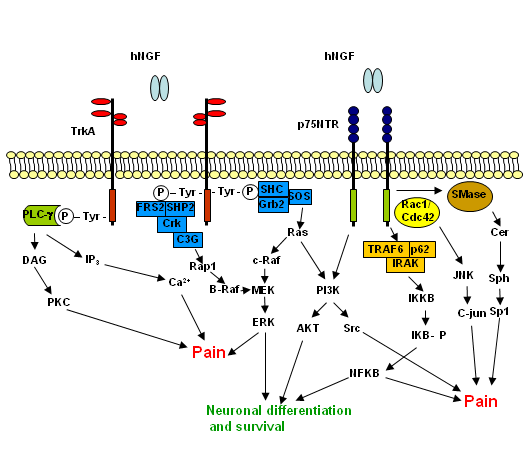

Supplement: Figure S2 — hNGF activation of TrkA and p75 NTR and their associated intracellular signaling pathways. The cartoon illustrates in a schematic manner the activation of TrkA and p75NTR by hNGF and the main downstream signaling pathways. As shown, the signaling streams leading to pain or to survival and growth/differentiation involve largely distinct signaling molecules, downstream of TrkA and p75NTR. Modified from Nicol and Vasko [25]. (TIF) [file pone.0017321.s007.tif]

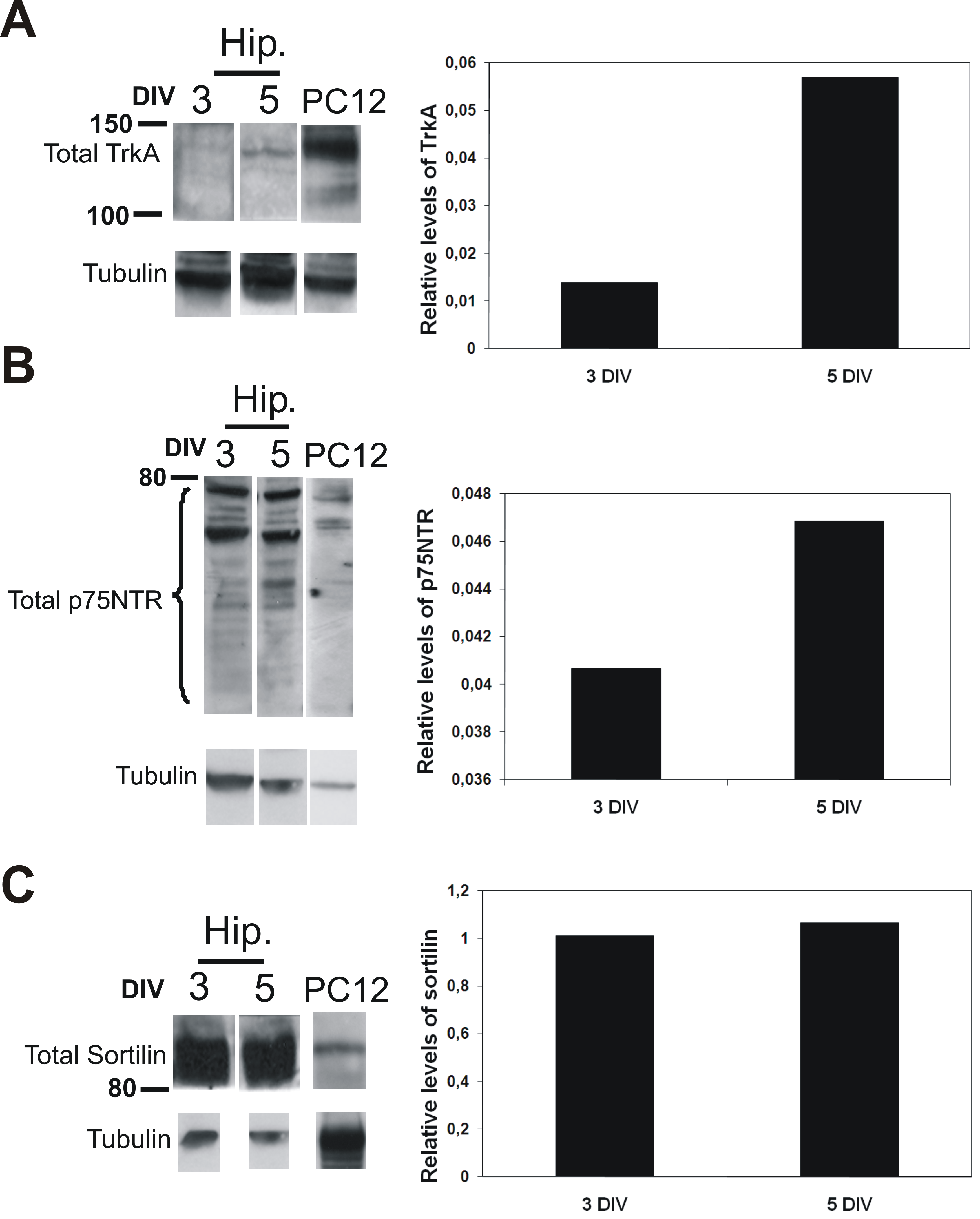

Supplement: Figure S3 — Expression of NGF receptors TrkA, P75NTR and sortilin in hippocampal neurons. Western blot and densitometric analysis of (A) pTrkA (Y490) (B) p75NTR and (C) sortilin in cell extracts from hippocampal cells after 3 and 5 days of culture compared to PC12 cells. Hippocampal cells were stimulated with 4 nM NGF. (TIF) [file pone.0017321.s008.tif]

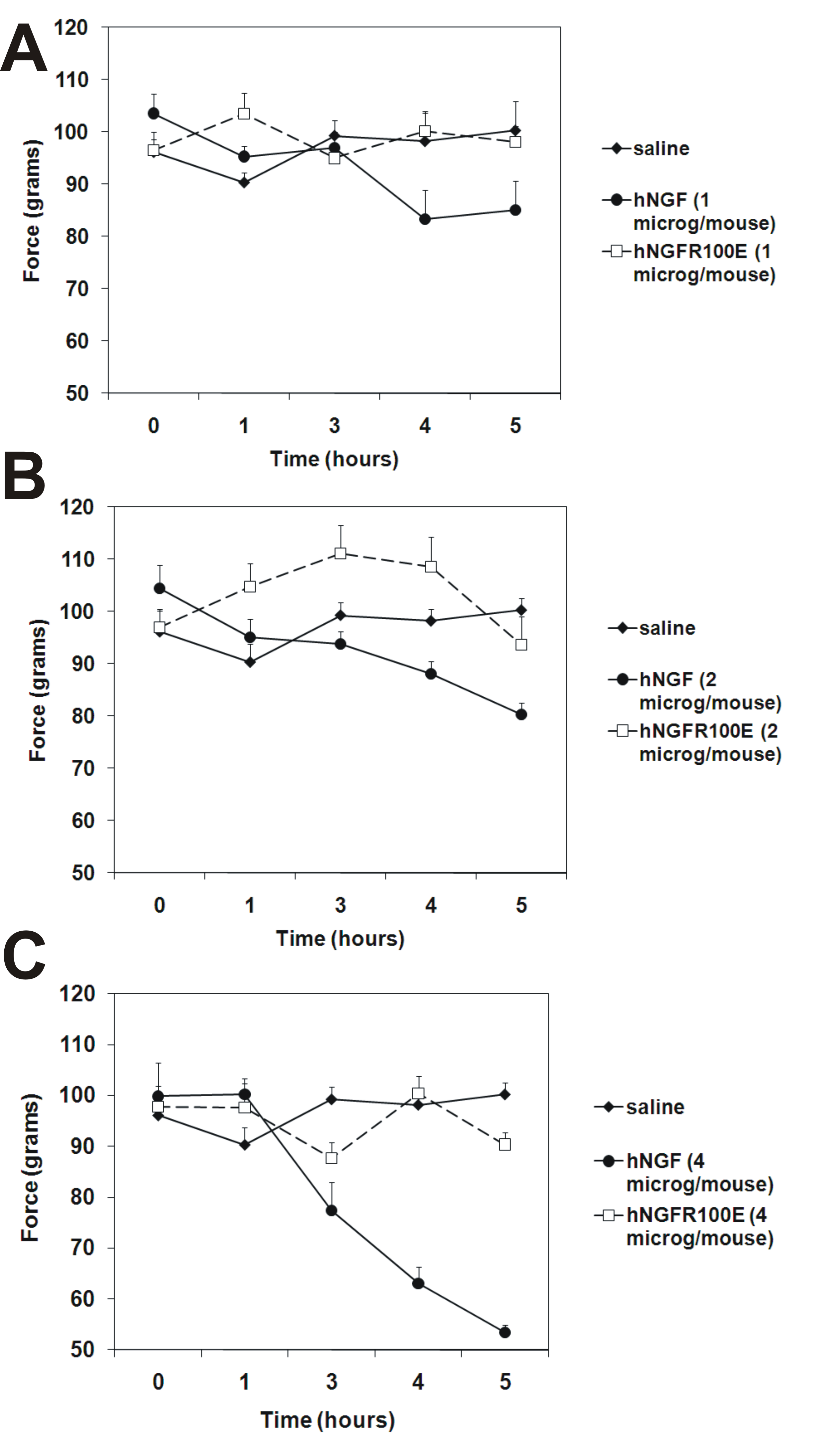

Supplement: Figure S4 — Time course of dose-dependent nociceptive response triggered by hNGF muteins. (A) 1 µg/mouse; (B) 2 µg/mouse and (C) 4 µg/mouse. At all doses and time points hNGFR100E does not induce pain. Points are the mean of the percentage derived from the ratio between ipsilateral vs controlateral measures ± s.e.m. (TIF) [file pone.0017321.s009.tif]
